# Supplementary material for: Discovery and Comprehensive Characterization of Novel Circular RNAs of the Apoptosis-Related BOK Gene in Human Ovarian and Prostate Cancer Cells, Using Nanopore Sequencing
Source: Noncoding RNA. 2023 Sep 24;9(5):57. doi: 10.3390/ncrna9050057 (PMC10609399; doi:10.3390/ncrna9050057)
Supplement: Supplementary file 1 [file ncrna-09-00057-s001.zip › Annotated reads.pdf]

Annotated representative reads

Note: All nested PCR primers are highlighted in yellow.

>40f50102-7ced-4302-b9b3-eaa885fef08a → circ-BOK-1 in MDAH-2774 (priming from Exon 4)

TGGCTGGCCACATCTTCTCTGCAGGCATCACGTGGGGCAAGGTGGTGTCCCCTGTATGCGGTGGCCGCGGGGCTGGCCG
TGGACTGTGTGAGGCAGGCCCAGCCTGCCATGGTCCACGCCCTCGTGGACTGCCTGGGGAGTTCGTGCGCAAGACCCTG
GCAACCTGGCTGCGGAGACGCGGCGGATGGACTGATGTCCTCAAGTGTGTGGTCAGCACAGACCCTGGCCTCCCGCTCC
CACTGGCTGGTGGCTGCACTCTGCGGCTTCGGCCTTCCTGAAGGCTGCCTTCTTCGTGCTGCTGCCAGAGATGAGCTGC
CCACCTGGCAGTGGCCGCAGCCTGGCCTCTGGGCCCAACGCAGGAAGGCCCTCAGCACCCGAACACATCTTCCTCCTCC
CCACCGAGCCTGGTGCCTCTAACCCTCGGAGACCCCTAAGCCCCACCGTCTCCAAGGCGATGAGCTGGAGATGATCCGG
CCCAGCGTCTACCGCAACGTGGC

>e57925de-d209-4fb2-b854-e51efd1e79d1 → circ-BOK-2 in Ishikawa (priming from Exon 4)

TGGCTGGCCACATCTTCTCCTTGCAGACTGATGTCCTCAAGTGTGTGGTCAGCACAGACCCTGGCCTCCGCTCCCACTG
GCTGGTGGCTGCACTCTGCAGCTTCGTGCGCTTCCTGAAGGCTGCCTTCTTCGTGCTGCTGCCAGAGAGATGATCCGGC
CCAGCGTCTACCGCAACGTGGC

>b0aefb15-0a92-46e5-a22f-813cae5ffadc → circ-BOK-3 in LNCaP (priming from Exon 5)

GCAAGACCCTGGCAACCTGGCTGCGGAGACGCGGCGGATGGACTGATGTCCTCAAGTGTGTGGTCAGCACAGACCCTGG
CCTCCGCTCCCACTGGCTGGTGGCTGCACTCCGCGGCTTCGGCCGCTTCCTGAAGGCTGCCTTCTTCGTGCTGCTGCCA
GAGAGATGAGCTGCCCACCTGGCAGTGGCCGCAGCCTGGCCCTCTGGGCCCAACGCAGGAGGCCCTCAGCACCCGAACA
CATCTTCTCTGCAGGCATCACGTGGGGCAAGGTGGTGTCCCTGTATGC

>fddfbaea-2d68-4b0d-9418-edaec2e4adf7 → circ-BOK-4 in Ishikawa (priming from Exon 4)

TGGCTGGCCACATCTTCTCTGCAGACTGATGTCCTCAAGTGTGTGGTCAGCACAGACCCTGGCCTCCGCTCCCACTGGC
TGGTGGCTGCACTCTGCAGCTTCGGCCGCTTCCTGAAGGCTGCCTTCTTCGTGCTGCTGCGCCTGGGCGATGAGCTGGA
GATGATCCGGCCAACGCGTCTACCGCAACGTGGC

>72967aa0-9547-40c0-86c9-b4d4ac38e8e2 → circ-BOK-5 in MDAH-2774 (priming from Exon 6)

GAGATGAGCTGCCCACCTGGCAGTGGCCAGCCTGGCCCTCTGGGCCCAACGCAGGAGGCCCTCAGCACCCGAACACATC
TTCCTCCTCCCCACCCGAGCCTGGAGCCTCTAACCTCGGAGACCCCTAAGCCCCCGTTCCTCCGAGGCCCCCCATTATG
GAAACTGCGCCCACCGGGCTGAGGTAGCCAAAACCTCAGTATTCGTTGGTGCTGTGAGGACATCTGGGGGTGCTCGGTT
CTGGGGTCACACACGGTCCACTGGCTGCCCCACCGTCTCCAAGGCGATGAGCTGGAGATGATCCGGCCCAGCGTCTACC
GCAACGTGGCGCGTCAGCTGCACATCTCCCTGCAGTCTGAGCCTGTGGTGACCGATGGGCCGTTCCTGGCCGTGGCTGG
CCACATCTTCTCTGCAGGCATCACGTGGGGCAAGGTGGTGTCCCCTGCGGTGGCCGCGGGGCTGGCCGTGGACTGTGTG
AGGCAGGCCCAGCCTGCCATGGTCCACGCCCTCGTGGACTGCCTGGGGGAGTTCGTGCGCAAGACCCTGCCGCCTGGCT
GCGGAGACGCGGCAGATGGACTGATGTCCTCAAGTGTGTGGTCAGC

>ae1e242f-2394-4e1c-96aa-e4f4b8f45fd3 → circ-BOK-6 in ES-2 (priming from Exon 6)

GAGATGAGCTGCCCACCTGGCAGTGGCCGCAGCCTGGCCCTCTGGGCCCAACGCAGGAGGCCCTCAGCACCCGAACACA
TCTTCCTCCTCCCCACCCGAGCCTGGAGCACTCTAACCCTCGGAGACCCCCTAAGCCCCCGTTCCTCCGCAGACCAGGC
CCTGGAAGGGGTGAGTGGGGAGGGGCTTTCCTGAGCCTGGAGCTGGGCTTTGGGGCAGCCTGCGACCCTCCCCGCTTGT
GTCCCTTCTCCTGTGATCTCTGTGTTTTCCCTTTTCTTTCTGGGGCCAGGAAGTCAGGGTCAACTCCCAGGCCTCAGAT
GCAGGGGCCCAGAACACCTGCTCT**TGTGACCCCACACAG**GCGATGAGCTGGAGATGATCCGGCCCAGCGTCTACCGCAA
CGTGGCGCGTCAGCTGCACATCTCCCTGCAGTCTGAGCCTGTGGTGACCGATGCGTTCCTGGCCGTGGCTGGCCACATC
TTCTCTGCAGGCATCACGTGGGGCAAGGTGGTGTCCCTGTATGCGGTGGCCGCGGGAGCTGGCCGTGGACTGTGTGAGG

CAAGCCCAGCCTGCCATGGTCACGTCCTCGTGGACTGCCTGGGGAGTTCGTGCGCAAGACCCTGGCAGCCTGGCTGCGG
AGACGGCGGATGGACTGATAAATGTCCTCAAGTGTGTGGTCAGC

>5910e378-b0e4-4b28-b8be-58baa5bc431f → circ-BOK-7 in ES-2 (priming from Exon 5)
GCAAGACCCTGGCAACCTGGCTGCGGAGACGCGGCGGATGGACTGATGTCCTCAAGTGTGTGGTCAGCACAGACCCTGG
CCTCCGCTCCCACTGGCTGGTGGCTGCACTCTGCAGCTTCGGCCGCTTCCTGAAGGCTGCCTTCTTCGTGCTGCTGAGC
CTGTGGTGACCGATGCGTTCCTGGCCGTGGCTGGCCACATCTTCTCTGCAGGCATCACGTGGGGCAAGGTGGTGTCCCT
GTATGC

>49b2f77f-988a-4045-af97-0b7c17e0cc40 → circ-BOK-8 in LNCaP (priming from Exon 4)
TGGCTGGCCACATCTTCTCTGCAGGCATCACGTGGGGCAAGGTGGTGTCCCTGTATGCGGTGGCCGCGGGGCTGCTGCG
CCTGGGCGATGAGCTGGAGATGATCGGCCCAGCGTCTACCGCAACGTGGC

>06b36cf2-0bf7-47fd-b447-5fc15140a3ec → circ-BOK-8 in MDAH-2774 (priming from Exon 4)
TGGCTGGCCACATCTTCTCCTGCAGGCATCACGTGGGGCAAGGTGGTGTCCCTGTATGCGGTGGCCGCGGGGCTGCTGC
GCCTGGGCGATGAGCTGGGAGATGATCCGGCCCAGCGTCTACCGCAACGTGGC

>064a6d7b-9bea-46f5-9f5e-f55172efb4cc → circ-BOK-9 in Ishikawa (priming from Exon 4)
TGGCTGGCCACATCTTCTCTCTGCAG**GTATGCCCAGCCTGCCCGTCCCATGGACCTCGGGGAGGGATCCAGGGTCTGT**
GGCTCAGGACAGCGGCAACCAGCCCAGGTGGGAGCGTGGCGGTGCGGGTGGCCCAGGACAGCAGCTCAGCCTCTGCATC
TGAGGTGGTCCCTGAGCCATGCTGAAGGCCTGAGCCGGGCTCTCGGGCTGTCCCCTGCTCTCACCCCAATGCTTTCTCT
GCCCAGCCCCTCCCCACGTCGGGCTCCTGCTCTCACCCAGTGCTTTCTCTGCTGACCCCTCCCCGCCAGGGTCTGGCCG
TCACCTGGTCCCCTCCTGCTCTGGGCAGCTCGGCTGCCTGTCGGCTGTGAGTCACCAGCAGGCACGACCCACGCTCTCG
CAGGATTCCCGCCCCACCGGCTTGGCCGGCTAGGCTTAGGGGCTGGCTCGTCAGAGGGGCAGGACTGGGGGCGCCTTCA
ATACTTCCTTGGTCTTCTGGTCCCTAGGGGCCAAGGTTGGGGGATGGCCCAGGGAGGTCAGATGGGGTCGAAATTAGGT
GGGAATCAGACAAGTGAGGAGCAGGCTGGAATTCAGCATAGTCAGGTGGGGTCAGGTGCAGGGTGTGCTTGCCCAAGCC
AGTCGTGTCCCAGGAAGCTGGGACGTGGGCTCTTCTCACCTGCTCTTGTGACCACACAGGCGATGAGCTGGAGTGAGTC
CGACGCGTCTACCGCAACGTGGC

>4eabdaf0-1f90-4da1-96c6-23c466d44f10 → circ-BOK-10 in LNCaP (priming from Exon 6)
GAGATGAGCTGCCCACCTGGCAGTGGCCGCAGCCGGCCTCTGGGCCCAACGCAGGAGGCCCTCAGCACCCGAACATCTT
CCTCCTCCCCACCCGAGCCTGGAGCACTCTAACCCTCGGAGACACAAGCCCCACGTTCTCCGCAGACCCAGGCCCTCCG
GAAGGGACTGATGTCCTCCAAGTGTGTGGTCAGC

>e16bb921-25bd-4670-b7c6-2ed50d7509be → circ-BOK-10 in OVCAR-3 (priming from Exon 6)
GAGATGAGCTGCCCACCTGGCAGTGGCCGCAGCCTGGCCCTCTGGGCCCAACGCAGGAGGCCCTCAGCACCCGGCTGCA
TCTTCCTCCTCCCCACCCGAGCCTGGACACTCTAACCCTCGGAGACCCCCTAAGCCCCGTTCCTCCACCAGACCCAGGC
CCTCCGGAAGGGACTGATGTCCTCCAAGTGTGTGGTCAGC

>0e49396b-21a7-440d-96cc-c6ada579a481 → circ-BOK-11 in MDAH-2774 (priming from Exon 5)
GCAAGACCCTGGCAACCTGGCTGCGGAGACGCGGCGGATGGACTGATGTCCTCAAGTGTGTGGTCAGCACAGACCCTGG
CCTCCGCTCCCACTGGCTGGTGGCTGCACATCTCCCTGCAGTCTGAGCCTGTGGTGACCGATGCGTTCCTGGCCGTGGC
TGGCCACATCTTCTCTGCAGGCATCACGTGGGGCAAGGTGGTGTCCCTGTATGC

>3e70100c-1f67-4988-af52-0cbcb027d1c3 → circ-BOK-12 in Ishikawa (priming from Exon 5)
GCAAGACCCTGGCAACCTGGCTGCGGAGACGCGGCGGATGG**GTGAGCGCCTGAGTGCCCGTGTGGGTGAGAGAGGCTGC**
TCCCATCTCAGTTCAGCCTCAGCAGGTTAGGGGTCTGGGAGGCAGTTTCTCTGCAGGCCTCAGCTTCTTTATGTGTAAA

ATGTGGACGCCAGGCCTGTTGGCCAGGAACAGGCAGCCCAGGGTGCCACCCGTGAGGTCACTGGCTGCTCACTCCATCT
GCTGCTGCCCTCAGTTCCTCGCCTCACCCCACGTTGTCCCTGCGGCCTCCTGCACAGACGTGGGCTGGGCAGGTGTGCA
GTGGAGGCCGAGAAACCTGCCTGCCCAAGAACTGCCTGACAGGCATCCTGTCAGGGAGCAGTCCCCGCAGGACGGTCCC
TGGTCATTAGCTGGACGGCAAGCAGGACAGCGGCTCAGAGGAGCCCAGCCCCTTCCTTAAGTGCTCCCGTGGCCGGGG
ATTCAAGTCCTGATCTTGACCATCTCTCTCCCTGCAGGCATCACGTGGGGCAAGGTGGTGTCCCTGTATGC

>8b99225a-1324-4766-8216-b537e5c6f3d6 → circ-BOK-13 in PC-3 (priming from Exon 5)
GCAAGACCCTGGCAACCTACCGGCTGCGGAGACGCGGCAGGATGGACTGATGTCCTCAGGTGTGTGGTCAGCACAGACC
CTGGCCTCCGCGCTCCCACTGGCTGGTGGCTGCCTCTGCAGCTCGGCGCTACCGAGCTGCACTTCTTCCGTGCTGCTGC
AGAGAGATGAGCTGGAGATGATCCGGCCCAGCGTCTACCGCAACGTGGCGCGTCAGCTGCACATCTCCCTGCAGTCCTG
AGCCTGTGGTGACCGATGCGTTCCTGGCCGTGGCTGGCCACATCTTCTCTGCAGGCATCACGTGGGGCAAGGTGGTGTC
CCTGCATGC

>2d9ee028-290f-4f2e-a0b0-85034fe52be8 → circ-BOK-13 in OVCAR-3 (priming from Exon 5)
GCAAGACCTGGCAACCTGGCTGCGGAGACGCGGCGGATGGACTGATGTCCTCAAGTGTGTGGTCAGCACAGACCCTGGC
CTCCGCTCCCGCTGGCTGTGGCTGCACTCTGCAGCTGTGACCGCTTCCTGAAGGCTACCTTCTTGTGCTGCTGCCAGAG
AGATGAGCTGGAGATGATCCGGCCCAGCGTCTACCGCAACGTGGCGTCAGCTGCACATCTCCCTGCAGTCTGAGCCTGT
GGTGACCGATGCGTTCTGACCGTGGCTGGCCACATCTTCTCTGCAGGCATCACGTGGAGCAAGGTGGTGTCCCTGTATG
C

>e3b87b06-ed07-484b-9e12-18e24f2635db → circ-BOK-14 in OVCAR-3 (priming from Exon 4)
TGGCTGGCCACATCTTCTCCTGCAGGCATCACGTGGGGCAAGGTGGTGTCCTGGCAATACGGTGGCCGCGGGGCTGGCC
GTGGACTGTGTGAGGCAGGCCAACCTGCCATAGTCCACGCCCTCGTGGACTGCCTGGGGGATTCGTGCGCGAGACCCGC
AGCAGCCTGGCTGCGGTGTGCGCGGTGCTGCTGCGCCTGGGCGATGAGCTGGAGATGATCCGGCCCAGCGTCTACCGCA
ACGTGGC

>c4d99b17-1141-4a39-8bf3-8b1df1b2f971 → circ-BOK-14 in OVCAR-3 (priming from Exon 5)
GCAAGACCTGGCGGCCTGGCTGCGGTGTGCCCGGTGCTGCTGCGCCTGGGCGATGAGCTGGAGATGATCCAGCCCGGCG
TCTACCTGACATGGCGCGTCAGCTGCACATCTCCCTGCAGTCTGAGCCTGTGGTGACCGATGCGTTCCTGGCCGTGGCT
GGCCACATCTTCTCTGCAGGCATCACGTGGGGCAAGGTGGTGTCCCTGTATGC

>369a3a97-5652-4b27-a437-4730d2d51642 → circ-BOK-15 in MDAH-2774 (priming from Exon 6)
GAGATGAGCTGCCCACCTGGCAGTGGCCGCAGCCTGGCCCTCTGGGCCCAACGCAGGAGGCCCTCAGCAC**AGAAACCCC**
AAGACTTCAAGTCCACCTCCTGCTGTCTTCCTCACATGGAGTCTGGCATGGGGCTGGGCACTGTGTGCCCAGTGGGCGA
CGACACTTTGAGCAACGCCGAGACCACAGGTCCAGGCTGCAGGGAAAGCCTTCATGCTGTTAGCAGTGAAGGGAATAAG
AGAATTGGACGTTCATTCACTTCCCAGAAGGAACTTCAGAGTCCAGGAGTCAAAGTAGGCTTCCAGAGGAAACGCAGTC
CCTGCTGTTGTTCCAGGCAGAGTCTGAATGAAGCTGGGTTTGCCCCTTCCTGGTAGCCCAGGCTCAAGGCCCCACAGGA
GGATGGCTTGACATACCTGTAGCCCCCTGGGGGGCAGCAGGCAGGTGGCCGAGGGAGCTGGCACCCTGGTCTGGATGTC
TGGAGCCCCAGCGAGGGCCATGAAGGCCTCTCTCCTGCTCGGCAGGGGCCCTCACGGCCGGCCTGCCTGGCTGCCATCT
GTCCCACACCTCCCGTGCCTCTGGCCCTTCACTGTCTAGGAATGTGCGGCATGGTCTGGTCTTGTGCTGGAGCCTTCAG
TCCAGGCCACAGACCTCCTTCCCGAACAGTCTCCTGCATTCCCTTCATGCAATTTTGCTGATATTCTGGTGCACAGTGC
TAGGGAGCCTGGCAGTGCTGGGGGCCCCTTGGCGGTGCTGGCTCTGCTTTCTAACGGTCTCCCTCTTCCCTCCCAGACT
GATGTCCTCAAGTGTGTGGTCAGC

>b74408fb-e4bb-4b0f-8b7c-62e1fa9eb36f → circ-BOK-16 in ES-2 (priming from Exon 4)
TGGCTGGCCACATCTTCTCTGCAG**GTATGCAGCCCCTGCCCGTCCCATGGACCTCAGGGAGGGATCCGGGGTCTGTGG
CTCAGGCTCACAGGACCCCACGAGCTGGCCCCAAGCCAGTCGTGTCCCAGGAAGCTGGGACGTGGGCTGCCTCTCACCT
GCTCTTGTGACCACACAG**GCGATGAGCTGGAGATGATCCGGCCCAGCGTCTACCGCAACGTGGC

>42b8ab7c-c9e5-4683-a083-82c95781e135 → circ-BOK-17 in DU 145 (priming from Exon 5)
GCAAGACCCTGGCAACCTGGCTGCGGAGACGCGGTGCTGCTGCGCCTGGATTCTTGCTGTTACTGGCCGAGATCCCGCC
CGCTGCCCTCTGGATTCCAGGCCTCCGAGGCCCCCATTGGAAACTGCGCCCACCGGGCTGGGTAGCCAAAACCTCAGTA
TTCGTTGGTGCTGTGAGGACATCTGGGGGTGCTCGGTTCTGGGGTCACACACGGTCCACTGGCTGCCCCCACCGTCTCC
AAGGCGATGAGCTGGAGATGATCCGGCCCAGCGTCTACCGCAACGTGGCGCGTCAGCTGCACATCTCCCTGCAGTCTGA
GCCTGTGGTGACCGATGCGTTCCTGGCCGTGGCTGTACATCTTCTCTGCAGGCATCACGTGGGGCAAGGTGGTGTCCCT
GTATGC

>3536c624-2557-4192-8006-3894e7532c42 (shown as reverse complement) → circ-BOK-18 in PC-3 (priming from Exon 5)
GCAAGACCCTGGCAACCTGGCTGCGGAGACGCGGCGGATGGACTGATGTCCTCAAGTGTGTGGTCAGCATGGCTGGCCA
TCTTCTCTGCAGGCATCACGTGGGGCAAGGTGGTGTCCCTGTATGC

>0e35b3ea-d741-4412-a035-dcdfbca3d251 (shown as reverse complement) → circ-BOK-19 in MDAH-2774 (priming from Exon 5)
GCAAGACCTGGCAACCTGGCTGGGTGGAGACGCGGCGGATGGACTGATGTCCTCAAGTGTGTGGTCAGTACAGACCCTG
GCCTCCGCTCCCACTGGCTGGTGGCTGCACTGCAGCTTCGGCCGTTTCTGAAGGCTGCCTTCTTCGTGCTGCTGCCAGA
GAGAAGGATGCCCACCTGGCAGTGGCCGCAGCCTTCGTCCTCTGGGCCCAACGCAGGAGGCCCTCAGCACCCGAACACA
TCTTCCTCCTCCCCACCCGAGCCTGGAGCACTCTAACCCTCGAATCCCCCTAAGCCCCGTTCCTCCGCAGACCCAGGCC
CTCCGGAAGGGGCATCACGTGGAGCAAGGTGGTGTCCCTGTATGC

>8d5a049f-eafe-4357-9e0b-b02717755a1a (shown as reverse complement) → circ-BOK-19 in MDAH-2774 (priming from Exon 6)
GAGATGAGCTGCCCACCTGGCAGTGGCCGCAGCCCGGCCCTCTGGGCCCAACGCGGGAGGCCCTCAGCACCCGAACACA
TCTTCCTCCTCCCCACCCGAGCCTGAGCACTCTAACCCCTCGGAGACCCCTAAGCCCCGTTCCTCCGCAGACCCAGGCC
CTCCGAAGGGGCATCACGGGAGGGGCAGGTGTCCCTGTATGCGGTGGCCGCGGGGCTGGCCGTGGACTGTGAGGCAGGC
CCAGCCTGCCATGGTCCACGCCCTCGTGGACTGGCTCGGGAGTTCCAGGCAAGACCCCGGCAACCTGGCTGCGGGAGAC
GCGGCGGATGGACTGATGTCCTCAAGTGTGTGGTCAGC

>ce685726-dd19-4da4-b50b-387c1567c017 → circ-BOK-20 in Ishikawa (priming from Exon 5)
GCAAGACCCTGGCAACCTGGCTGCGGAGACGCGGCGGATGGACTGATGTCCTCAAGTGTGTGGTCAGCACAGACCCTGG
CCTCCGCTCCCACTGGCTGGTGGCTGCACTCTGCAGGCATCACGTGGGGCAAGGTGGTGTCCCTGTATGC

>ba319470-8dd1-4a22-a7d3-ce13d4538656 → circ-BOK-20 in OVCAR-3 (priming from Exon 5)
GCAAGACCCTGGCAACCTGGCTGCGGAGACGCGGCGGATGGACTGATGTCCTCAAGTGTGTGGTCAGCACAGACCCTGG
CCTCCGCTCCCACTGGCTGGTGGCTGCACTCTGCAGGCATCACGTGGGGCAAGGTGGTGTCCCTGTATGC

>392a6918-65c1-4f0a-a355-5201b5b51b89 → circ-BOK-21 in LNCaP (priming from Exon 6)
GAGATGAGCTGCCCACCTGGCAGTGTACAACCTGGCCTCTGGGCCCAGCGCAGAGGCCCTCAGCACCCGAACACATCTT
CCTCCTCCACCCGAGCCTGGAGCACTCTAACCCTCGGAGACGCGGCGGATGGACTGATGTCCTCAAGGAGTGTGTGGTC
AGC

>0039de89-8f13-4469-9a87-f5fc65cbf6c5 → circ-BOK-21 in MDAH-2774 (priming from Exon 6)
GAGATGAGCTGCCCACCCTGGCAGTGGCCGCAGCCTGGCCCTCTGGGCCCAACGCAGGGAGGCCCTCAGCACCGAACAC
ATCTTCCTCCCTCCCCACCCGAGCCTGGGTACTCTAACCCTCGGAGACGCGGCGGATGGACTGATGTCCTCAAGTGTGT
GGTCAGC

>24c16eab-bec6-4c80-be36-4b6f026c04a5 → circ-BOK-22 in PC-3 (priming from Exon 4)
TGGCTGGCCACATCTTCTCTTGCAGGCATCACGTGGGAGCGGGTGCCAGGCTACTGTATGCGGTGGCCGCGGGGCTGGC
CGTGGACTGTGTGAGGCAGGCCAGCCTGCCATGGTCACGCCCTCGTGGACTGCCTGGCTGAGGAGGTGTGCGCGGTGCT
GCTGCGCCTGGGCGATGAGCTGGAGATGATCCGGCCCAGCGTCTACCGCAACGTGGC

>693b2039-411d-4723-bec3-0209ed9a6b04 → circ-BOK-23 in PC-3 (priming from Exon 6)
GAGATGAGCTGCCCACCTGGCAGTGGCCGCAGCCTGGCCCTCTGGGCCCAACGCAGGGAAGGCCCTCAGCGCCCGAACT
CCCCAGTCCACTTTTCGTGGACTGCCTGGGGGAGTTCTCTTGCAAGACCCTGGCAACCTGGCTGCGGAACGCGGCGGAT
GGACTGATGTCCTCAGATGTGTGGTCAGC
