# Supplementary material for: Discovery and Comprehensive Characterization of Novel Circular RNAs of the Apoptosis-Related BOK Gene in Human Ovarian and Prostate Cancer Cells, Using Nanopore Sequencing
Source: Noncoding RNA. 2023 Sep 24;9(5):57. doi: 10.3390/ncrna9050057 (PMC10609399; doi:10.3390/ncrna9050057)
Supplement: Supplementary file 1 [file ncrna-09-00057-s001.zip › BOK circRNA sequences.pdf]

***BOK* circRNA sequences**

Note: The unique yellow-highlighted “A” appearing in circ-BOK-12 is “G” in the genomic *BOK* sequence.

>circ-BOK-1
CCCCACCGTCTCCAAGGCGATGAGCTGGAGATGATCCGGCCCAGCGTCTACCGCAACGTGGCGCGTCAGC
TGCACATCTCCCTGCAGTCTGAGCCTGTGGTGACCGATGCGTTCCTGGCCGTGGCTGGCCACATCTTCTC
TGCAGGCATCACGTGGGGCAAGGTGGTGTCCCTGTATGCGGTGGCCGCGGGGCTGGCCGTGGACTGTGTG
AGGCAGGCCCAGCCTGCCATGGTCCACGCCCTCGTGGACTGCCTGGGGGAGTTCGTGCGCAAGACCCTGG
CAACCTGGCTGCGGAGACGCGGCGGATGGACTGATGTCCTCAAGTGTGTGGTCAGCACAGACCCTGGCCT
CCGCTCCCACTGGCTGGTGGCTGCACTCTGCAGCTTCGGCCGCTTCCTGAAGGCTGCCTTCTTCGTGCTG
CTGCCAGAGAGATGAGCTGCCCACCTGGCAGTGGCCGCAGCCTGGCCCTCTGGGCCCAACGCAGGAGGCC
CTCAGCACCCGAACACATCTTCCTCCTCCCCACCCGAGCCTGGAGCACTCTAACCCTCGGAGACCCCCTA
AG

>circ-BOK-2
GATGATCCGGCCCAGCGTCTACCGCAACGTGGCGCGTCAGCTGCACATCTCCCTGCAGTCTGAGCCTGTG
GTGACCGATGCGTTCCTGGCCGTGGCTGGCCACATCTTCTCTGCAGACTGATGTCCTCAAGTGTGTGGTC
AGCACAGACCCTGGCCTCCGCTCCCACTGGCTGGTGGCTGCACTCTGCAGCTTCGGCCGCTTCCTGAAGG
CTGCCTTCTTCGTGCTGCTGCCAGAGA

>circ-BOK-3
CACATCTTCTCTGCAGGCATCACGTGGGGCAAGGTGGTGTCCCTGTATGCGGTGGCCGCGGGGCTGGCCG
TGGACTGTGTGAGGCAGGCCCAGCCTGCCATGGTCCACGCCCTCGTGGACTGCCTGGGGGAGTTCGTGCG
CAAGACCCTGGCAACCTGGCTGCGGAGACGCGGCGGATGGACTGATGTCCTCAAGTGTGTGGTCAGCACA
GACCCTGGCCTCCGCTCCCACTGGCTGGTGGCTGCACTCTGCAGCTTCGGCCGCTTCCTGAAGGCTGCCT
TCTTCGTGCTGCTGCCAGAGAGATGAGCTGCCCACCTGGCAGTGGCCGCAGCCTGGCCCTCTGGGCCCAA
CGCAGGAGGCCCTCAGCACCCGAA

>circ-BOK-4
CTGCGCCTGGGCGATGAGCTGGAGATGATCCGGCCCAGCGTCTACCGCAACGTGGCGCGTCAGCTGCACA
TCTCCCTGCAGTCTGAGCCTGTGGTGACCGATGCGTTCCTGGCCGTGGCTGGCCACATCTTCTCTGCAGA
CTGATGTCCTCAAGTGTGTGGTCAGCACAGACCCTGGCCTCCGCTCCCACTGGCTGGTGGCTGCACTCTG
CAGCTTCGGCCGCTTCCTGAAGGCTGCCTTCTTCGTGCTG

>circ-BOK-5
CCTCCGAGGCCCCCCCATTGGAAACTGCGCCCACCGGGCTGGGTAGCCAAAACCTCAGTATTCGTTGGTG
CTGTGAGGACATCTGGGGGTGCTCGGTTCTGGGGTCACACACGGTCCACTGGCTGCCCCCACCGTCTCCA
AGGCGATGAGCTGGAGATGATCCGGCCCAGCGTCTACCGCAACGTGGCGCGTCAGCTGCACATCTCCCTG
CAGTCTGAGCCTGTGGTGACCGATGCGTTCCTGGCCGTGGCTGGCCACATCTTCTCTGCAGGCATCACGT
GGGGCAAGGTGGTGTCCCTGTATGCGGTGGCCGCGGGGCTGGCCGTGGACTGTGTGAGGCAGGCCCAGCC
TGCCATGGTCCACGCCCTCGTGGACTGCCTGGGGGAGTTCGTGCGCAAGACCCTGGCAACCTGGCTGCGG
AGACGCGGCGGATGGACTGATGTCCTCAAGTGTGTGGTCAGCACAGACCCTGGCCTCCGCTCCCACTGGC

TGGTGGCTGCACTCTGCAGCTTCGGCCGCTTCCTGAAGGCTGCCTTCTTCGTGCTGCTGCCAGAGAGATG
AGCTGCCCACCTGGCAGTGGCCGCAGCCTGGCCCTCTGGGCCCAACGCAGGAGGCCCTCAGCACCCGAAC
ACATCTTCCTCCTCCCCACCCGAGCCTGGAGCACTCTAACCCTCGGAGACCCCCTAAGCCCCGTT

>circ-BOK-6

CACCTGCTCT**TGTGACCACACAG**GCGATGAGCTGGAGATGATCCGGCCCAGCGTCTACCGCAACGTGGCG
CGTCAGCTGCACATCTCCCTGCAGTCTGAGCCTGTGGTGACCGATGCGTTCCTGGCCGTGGCTGGCCACA
TCTTCTCTGCAGGCATCACGTGGGGCAAGGTGGTGTCCCTGTATGCGGTGGCCGCGGGGCTGGCCGTGGA
CTGTGTGAGGCAGGCCCAGCCTGCCATGGTCCACGCCCTCGTGGACTGCCTGGGGGAGTTCGTGCGCAAG
ACCCTGGCAACCTGGCTGCGGAGACGCGGCGGATGGACTGATGTCCTCAAGTGTGTGGTCAGCACAGACC
CTGGCCTCCGCTCCCACTGGCTGGTGGCTGCACTCTGCAGCTTCGGCCGCTTCCTGAAGGCTGCCTTCTT
CGTGCTGCTGCCAGAGAGATGAGCTGCCCACCTGGCAGTGGCCGCAGCCTGGCCCTCTGGGCCCAACGCA
GGAGGCCCTCAGCACCCGAACACATCTTCCTCCTCCCCACCCGAGCCTGGAGCACTCTAACCCTCGGAGA
CCCCCTAAGCCCCGTTCCTCCGCAGACCCAGGCCCTCCGGAAGGGGTGAGTGGGGAGGGGCTTTCCTGAG
CCTGGAGCTGGGCTTTGGGGCAGCCTGCGACCCTCCCCGCTTGTGTCCCTTCTCCTGTGATCTCTGTGTT
TTCCCTTTTCTTTCTGGGGCCAGGAAGTCAGGGTCAACTCCCAGGCCTCAGATGCAGGGGCCCAGAA

>circ-BOK-7

CTGAGCCTGTGGTGACCGATGCGTTCCTGGCCGTGGCTGGCCACATCTTCTCTGCAGGCATCACGTGGGG
CAAGGTGGTGTCCCTGTATGCGGTGGCCGCGGGGCTGGCCGTGGACTGTGTGAGGCAGGCCCAGCCTGCC
ATGGTCCACGCCCTCGTGGACTGCCTGGGGGAGTTCGTGCGCAAGACCCTGGCAACCTGGCTGCGGAGAC
GCGGCGGATGGACTGATGTCCTCAAGTGTGTGGTCAGCACAGACCCTGGCCTCCGCTCCCACTGGCTGGT
GGCTGCACTCTGCAGCTTCGGCCGCTTCCTGAAGGCTGCCTTCTTCGTGCTG

>circ-BOK-8

CTGCGCCTGGGCGATGAGCTGGAGATGATCCGGCCCAGCGTCTACCGCAACGTGGCGCGTCAGCTGCACA
TCTCCCTGCAGTCTGAGCCTGTGGTGACCGATGCGTTCCTGGCCGTGGCTGGCCACATCTTCTCTGCAGG
CATCACGTGGGGCAAGGTGGTGTCCCTGTATGCGGTGGCCGCGGGGCTG

>circ-BOK-9

G**ACAGCGGCCCAGCCAGCCCAGGTGGGAGCGTGGCAGTGCGGGTGGCCCAGGACAGCGGCTCAGCCTCTG**
CATCTGAGTGGTCCCTGAGCCATGCTAGGGCCTGAGCCGGGCTCTCGGGCTGTCCCCTGCTCTCACCCCA
ATGCTTTCTCTGCCCAGCCCCTCCCCACGTCGGGCTCCCCTGCTCTCACCCCAGTGCTTTCTCTGCCCAG
CCCCTCCCCACGTCGGGGTCTGGCCGTCTGCCTGGTCCCTCCTGCTCTGGGCAGCTCGGCTGCCTGTCGG
CTGTGAGTCACCAGCAGGCACGGCCCACGCTCTCGCAGGATTCCCGCCCCACCGGCTTGGCCGGCTAGGC
TTAGGGGCTGGCTCGTCAGAGGGGCGGGACTGGGGGCGCCTTCAGTACTTCCTTGGTCTTCTGGTCCCTA
GGGGCCAAGGTTGGGGGATGGCCCAAGGGAGGTCAGATGGGGTCAAGTGGAGGTGGGAATCAGACAAGTG
AGGAACAGGCTGGAATTCAAGCATGGTCAGGTGGGGGTCAGGTGCAGGGTGTGCCCCAAGCCAGTCGTGT
CCCAGGAAGCTGGGACGTGGGCTGCCTCTCACCTGCTCTTGTGACCACACAGGCGATGAGCTGGAGATGA
TCCGGCCCAGCGTCTACCGCAACGTGGCGCGTCAGCTGCACATCTCCCTGCAGTCTGAGCCTGTGGTGAC
CGATGCGTTCCTGGCCGTGGCTGGCCACATCTTCTCTGCAG**GTATGCCCAGCCTGCCCGTCCCATGGAC**
CTCAGGGAGGGATCCAGGTCTGTGGCTCAG

>circ-BOK-10

ACTGATGTCCTCAAGTGTGTGGTCAGCACAGACCCTGGCCTCCGCTCCCACTGGCTGGTGGCTGCACTCT
GCAGCTTCGGCCGCTTCCTGAAGGCTGCCTTCTTCGTGCTGCTGCCAGAGAGATGAGCTGCCCACCTGGC
AGTGGCCGCAGCCTGGCCCTCTGGGCCCAACGCAGGAGGCCCTCAGCACCCGAACACATCTTCCTCCTCC
CCACCCGAGCCTGGAGCACTCTAACCCTCGGAGACCCCCTAAGCCCCGTTCCTCCGCAGACCCAGGCCCT
CCGGAAGGG

>circ-BOK-11

GCTGCACATCTCCCTGCAGTCTGAGCCTGTGGTGACCGATGCGTTCCTGGCCGTGGCTGGCCACATCTTC
TCTGCAGGCATCACGTGGGGCAAGGTGGTGTCCCTGTATGCGGTGGCCGCGGGGCTGGCCGTGGACTGTG
TGAGGCAGGCCCAGCCTGCCATGGTCCACGCCCTCGTGGACTGCCTGGGGGAGTTCGTGCGCAAGACCCT
GGCAACCTGGCTGCGGAGACGCGGCGGATGGACTGATGTCCTCAAGTGTGTGGTCAGCACAGACCCTGGC
CTCCGCTCCCACTGGCTGGTG

>circ-BOK-12

AGGCTGCTCCCATCTCAGTTCAGCCTCAGCAGGTTAGGGGTCTGGGGAAGGCAGTTTCTCTGCGGGCCTC
AGCTTCTTTATGTGTAAAATGTGGACGCCAGGCCTGTTGTGGGAACAGGCAGCCCAGGGGTGCCACCCGT
GAGGTCACTGGCTGCTCACTCCATCTGCTGCTGCCCTCAGTTCCCTCGCCTCACCCCACGTTGTCCCTGC
GGCCTCCTGCACAGACGTGGGCTGGGCAGGTGTGCAGTGGAGGCCGAGAAACCTGCCTGCCCAAGAACTA
CCTGACAGGCATCCTGTCAGGGAGCGGTCCCCCCTTGGGACGGTCCCTGGTCATTAGCTGGACGGCAAGC
AGGACAGCGGCTCAGAGAGGCCCAGCCCCTTCCTTAAGTGCTCCCGTGGGCGGGATTCAAGTCCTGATCT
TGACCATCTCTCTCCCTGCAGGCATCACGTGGGGCAAGGTGGTGTCCCTGTATGCGGTGGCCGCGGGGCT
GGCCGTGGACTGTGTGAGGCAGGCCCAGCCTGCCATGGTCCACGCCCTCGTGGACTGCCTGGGGGAGTTC
GTGCGCAAGACCCTGGCAACCTGGCTGCGGAGACGCGGCGGATGG**GTGAGCGCCTGAGTGCCCGTGTGGG**
TGAGAG

>circ-BOK-13

GATGAGCTGGAGATGATCCGGCCCAGCGTCTACCGCAACGTGGCGCGTCAGCTGCACATCTCCCTGCAGT
CTGAGCCTGTGGTGACCGATGCGTTCCTGGCCGTGGCTGGCCACATCTTCTCTGCAGGCATCACGTGGGG
CAAGGTGGTGTCCCTGTATGCGGTGGCCGCGGGGCTGGCCGTGGACTGTGTGAGGCAGGCCCAGCCTGCC
ATGGTCCACGCCCTCGTGGACTGCCTGGGGGAGTTCGTGCGCAAGACCCTGGCAACCTGGCTGCGGAGAC
GCGGCGGATGGACTGATGTCCTCAAGTGTGTGGTCAGCACAGACCCTGGCCTCCGCTCCCACTGGCTGGT
GGCTGCACTCTGCAGCTTCGGCCGCTTCCTGAAGGCTGCCTTCTTCGTGCTGCTGCCAGAGA

>circ-BOK-14

GGTGTGCGCGGTGCTGCTGCGCCTGGGCGATGAGCTGGAGATGATCCGGCCCAGCGTCTACCGCAACGTG
GCGCGTCAGCTGCACATCTCCCTGCAGTCTGAGCCTGTGGTGACCGATGCGTTCCTGGCCGTGGCTGGCC
ACATCTTCTCTGCAGGCATCACGTGGGGCAAGGTGGTGTCCCTGTATGCGGTGGCCGCGGGGCTGGCCGT
GGACTGTGTGAGGCAGGCCCAGCCTGCCATGGTCCACGCCCTCGTGGACTGCCTGGGGGAGTTCGTGCGC
AAGACCCTGGCAACCTGGCTGC

>circ-BOK-15

CAC**AGAAACCCCAAGACTTCCGTCCCCTCCTGTCTTCCTCCTGTGGAGTCTGGCATGGGGCTGGGCACTG**
TGTGCCCAGTGGGCGACGACACTTTGAGCAACGCCAAGGCCACAGGTCCAGGCTGCAGGGAAAAGCCTTC

ATGTATTAGCAGGAAGTGAAGGGAATAAGAGAATTGGACGTTCATTCACTTCCCCAGAAGGAACTTCAGA
GTCCAGGGAGTCCGTGGGCTTCCGGAAACGCAGTCCCTGCTGTTGTTCCAGGCAGAGTCTGAATGAAGCT
GGGTTTGCCCCTTCCTGGTAGCCCAGGCTCCAAGGCCCCACAGGAGGATGACGGCGGCATACCTGTAGCC
CCTGGGGGGCAGCAGGCAGGTGGCCGAGGGGAGCTGGCACCCTGGTCTGGATGTCTGGAGCCCCAGCGAG
GGCCATGAAGGCCTCTCTCCTGCTCGGCAGGGGCCCTCCGGCCGGCCTGCCTGGCTGCCATCTGTCCCCA
CCTCCCGTGCCTCCTGGCCCTTCACACTGTCTGAATGTGCACGGCATCGGGTCTGGTCTTGTGTTGGAGC
CTTCAGTCCAGGCCACAGACCTCCTTCCCGAACAGTCTCCTGCATTCCCTTCATGCAATTTTGCTGATAT
TCTGGTGCACGGTGCTGGGGGGCCTGGCGGTGCTGGGGGCCTGGCGGTGCTGGCTCTGCTTTCTAACGGT
CTCCCTCTTCCCTCCCAGACTGATGTCCTCAAGTGTGTGGTCAGCACAGACCCTGGCCTCCGCTCCCACT
GGCTGGTGGCTGCACTCTGCAGCTTCGGCCGCTTCCTGAAGGCTGCCTTCTTCGTGCTGCTGCCAGAGAG
ATGAGCTGCCCACCTGGCAGTGGCCGCAGCCTGGCCCTCTGGGCCCAACGCAGGAGGCCCTCAG

>circ-BOK-16
GCCCCAAG**CCAGTCGTGTCCCAGGAAGCTGGGACGTGGGCTGCCTCTCACCTGCTCTTGTGACCACACAG**
GCGATGAGCTGGAGATGATCCGGCCCAGCGTCTACCGCAACGTGGCGCGTCAGCTGCACATCTCCCTGCA
GTCTGAGCCTGTGGTGACCGATGCGTTCCTGGCCGTGGCTGGCCACATCTTCTCTGCAG**GTATGCCCAGC**
CTGCCCGTCCCATGGGACCTCAGGGAGGGATCCAGGGTCTGTGGCTCAGGCTCACAGGGACCCCACGAGC
TG

>circ-BOK-17
GTGCTGCTGCGCCTGGATTCTTGCTGTTACTGGCCGAGATCCCGCCCGCTGCCCTCTGGATTCCGAGGCC
TCCGAGGCCCCCCCATTGGAAACTGCGCCCACCGGGCTGGGTAGCCAAAACCTCAGTATTCGTTGGTGCT
GTGAGGACATCTGGGGGTGCTCGGTTCTGGGGTCACACACGGTCCACTGGCTGCCCCCACCGTCTCCAAG
GCGATGAGCTGGAGATGATCCGGCCCAGCGTCTACCGCAACGTGGCGCGTCAGCTGCACATCTCCCTGCA
GTCTGAGCCTGTGGTGACCGATGCGTTCCTGGCCGTGGCTGGCCACATCTTCTCTGCAGGCATCACGTGG
GGCAAGGTGGTGTCCCTGTATGCGGTGGCCGCGGGGCTGGCCGTGGACTGTGTGAGGCAGGCCCAGCCTG
CCATGGTCCACGCCCTCGTGGACTGCCTGGGGGAGTTCGTGCGCAAGACCCTGGCAACCTGGCTGCGGAG
ACGCG

>circ-BOK-18
TGGCTGGCCACATCTTCTCTGCAGGCATCACGTGGGGCAAGGTGGTGTCCCTGTATGCGGTGGCCGCGGG
GCTGGCCGTGGACTGTGTGAGGCAGGCCCAGCCTGCCATGGTCCACGCCCTCGTGGACTGCCTGGGGGAG
TTCGTGCGCAAGACCCTGGCAACCTGGCTGCGGAGACGCGGCGGATGGACTGATGTCCTCAAGTGTGTGG
TCAGCA

>circ-BOK-19
GCATCACGTGGGGCAAGGTGGTGTCCCTGTATGCGGTGGCCGCGGGGCTGGCCGTGGACTGTGTGAGGCA
GGCCCAGCCTGCCATGGTCCACGCCCTCGTGGACTGCCTGGGGGAGTTCGTGCGCAAGACCCTGGCAACC
TGGCTGCGGAGACGCGGCGGATGGACTGATGTCCTCAAGTGTGTGGTCAGCACAGACCCTGGCCTCCGCT
CCCACTGGCTGGTGGCTGCACTCTGCAGCTTCGGCCGCTTCCTGAAGGCTGCCTTCTTCGTGCTGCTGCC
AGAGAGATGAGCTGCCCACCTGGCAGTGGCCGCAGCCTGGCCCTCTGGGCCCAACGCAGGAGGCCCTCAG
CACCCGAACACATCTTCCTCCTCCCCACCCGAGCCTGGAGCACTCTAACCCTCGGAGACCCCCTAAGCCC
CGTTCCTCCGCAGACCCAGGCCCTCCGGAAGGG

>circ-BOK-20

GCATCACGTGGGGCAAGGTGGTGTCCCTGTATGCGGTGGCCGCGGGGCTGGCCGTGGACTGTGTGAGGCA
GGCCCAGCCTGCCATGGTCCACGCCCTCGTGGACTGCCTGGGGGAGTTCGTGCGCAAGACCCTGGCAACC
TGGCTGCGGAGACGCGGCGGATGGACTGATGTCCTCAAGTGTGTGGTCAGCACAGACCCTGGCCTCCGCT
CCCACTGGCTGGTGGCTGCACTCTGCAG

>circ-BOK-21

CGGAGACGCGGCGGATGGACTGATGTCCTCAAGTGTGTGGTCAGCACAGACCCTGGCCTCCGCTCCCACT
GGCTGGTGGCTGCACTCTGCAGCTTCGGCCGCTTCCTGAAGGCTGCCTTCTTCGTGCTGCTGCCAGAGAG
ATGAGCTGCCCACCTGGCAGTGGCCGCAGCCTGGCCCTCTGGGCCCAACGCAGGAGGCCCTCAGCACCCG
AACACATCTTCCTCCTCCCCACCCGAGCCTGGAGCACTCTAACCCT

>circ-BOK-22

GCCTGGCTGAGGTGTGCGCGGTGCTGCTGCGCCTGGGCGATGAGCTGGAGATGATCCGGCCCAGCGTCTA
CCGCAACGTGGCGCGTCAGCTGCACATCTCCCTGCAGTCTGAGCCTGTGGTGACCGATGCGTTCCTGGCC
GTGGCTGGCCACATCTTCTCTGCAGGCATCACGTGGGGCAAGGTGGTGTCCCTGTATGCGGTGGCCGCGG
GGCTGGCCGTGGACTGTGTGAGGCAGGCCCAGCCTGCCATGGTCCACGCCCTCGTGGACT

>circ-BOK-23

GTCCACGCCCTCGTGGACTGCCTGGGGGAGTTCGTGCGCAAGACCCTGGCAACCTGGCTGCGGAGACGCG
GCGGATGGACTGATGTCCTCAAGTGTGTGGTCAGCACAGACCCTGGCCTCCGCTCCCACTGGCTGGTGGC
TGCACTCTGCAGCTTCGGCCGCTTCCTGAAGGCTGCCTTCTTCGTGCTGCTGCCAGAGAGATGAGCTGCC
CACCTGGCAGTGGCCGCAGCCTGGCCCTCTGGGCCCAACGCAGGAGGCCCTCAGCACCCGAACACATCTT
CCTCCTCCCCA
